# Principles of targeting endothelial cell metabolism to treat angiogenesis and endothelial cell dysfunction in disease

**DOI:** 10.15252/emmm.201404156

**Published:** 2014-07-25

**Authors:** Jermaine Goveia, Peter Stapor, Peter Carmeliet

**Affiliations:** 1 Laboratory of Angiogenesis and Neurovascular Link, Vesalius Research Center, Department of Oncology, University of LeuvenLeuven, Belgium; 2 Laboratory of Angiogenesis and Neurovascular Link, Vesalius Research Center, VIBLeuven, Belgium

**Keywords:** angiogenesis, endothelial cell dysfunction, metabolism

## Abstract

The endothelium is the orchestral conductor of blood vessel function. Pathological blood vessel formation (a process termed pathological angiogenesis) or the inability of endothelial cells (ECs) to perform their physiological function (a condition known as EC dysfunction) are defining features of various diseases. Therapeutic intervention to inhibit aberrant angiogenesis or ameliorate EC dysfunction could be beneficial in diseases such as cancer and cardiovascular disease, respectively, but current strategies have limited efficacy. Based on recent findings that pathological angiogenesis and EC dysfunction are accompanied by EC-specific metabolic alterations, targeting EC metabolism is emerging as a novel therapeutic strategy. Here, we review recent progress in our understanding of how EC metabolism is altered in disease and discuss potential metabolic targets and strategies to reverse EC dysfunction and inhibit pathological angiogenesis.

## Introduction

Blood vessels perform many functions that are critical for tissue homeostasis (Carmeliet, 2003). The endothelium, a single layer of endothelial cells (ECs) that lines the blood vessel lumen, controls vessel function. EC functions include the regulation of vascular tone and barrier, leukocyte trafficking, blood coagulation, nutrient and electrolyte uptake and neovascularization of hypoxic tissue, to name only a few (Cines *et al*, 1998; Pober *et al*, 2009; Potente *et al*, 2011). Many diseases are characterized by pathological blood vessel responses or formation. The inability of ECs to perform their physiological function (a condition termed EC dysfunction) contributes to cardiovascular disease and diabetes (Davignon & Ganz, 2004), whereas diseases such as cancer and age-related macula degeneration are characterized by new blood vessel formation (a process termed angiogenesis) (Carmeliet & Jain, 2011). Targeting ECs to prevent dysfunction or inhibit is potentially beneficial for a wide variety of diseases, but current treatment modalities, focusing primarily on growth factors, receptors, signaling molecules and others have limited efficacy or specificity (Bergers & Hanahan, 2008; Versari *et al*, 2009; Lee *et al*, 2012).

An emerging but understudied therapeutic target is EC metabolism. It has been long known that risk factors for cardiovascular disease (hypercholesterolemia, hypertension, dyslipidemia, diabetes, obesity and aging) cause EC-specific metabolic perturbations leading to EC dysfunction (Davignon & Ganz, 2004; Pober *et al*, 2009). Similarly, the links between EC metabolism and angiogenesis are apparent as angiogenic ECs migrate and proliferate in metabolically challenging environments such as hypoxic and nutrient-deprived tissue (Harjes *et al*, 2012). Moreover, the growth factor-induced switch from a quiescent to an angiogenic phenotype is mediated by important adaptations in EC energy metabolism (De Bock *et al*, 2013a,b; Schoors *et al*, 2014a,b). EC metabolic alterations are therefore not just innocent bystanders but mediate pathogenesis. In this review, we summarize existing data on the role of EC metabolism in mediating vascular disease and discuss how metabolism may be targeted for therapeutic benefit.

## General endothelial metabolism

**Figure 1:**
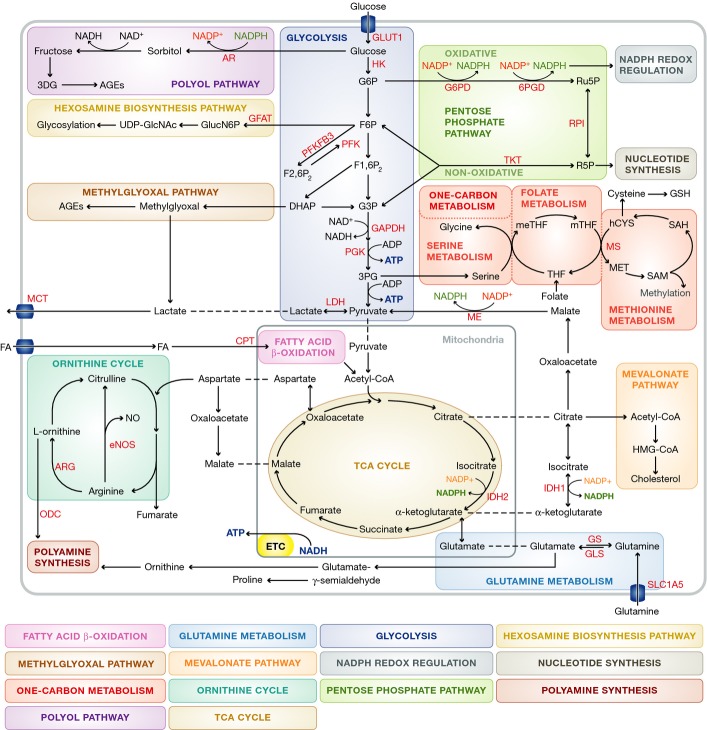
Overview of general EC metabolism For clarity, not all metabolites and enzymes of the depicted pathways are shown. Abbreviations: 3DG: 3-deoxyglucosone; 3PG: 3-phosphoglycerate; 6PGD: 6-phosphogluconate dehydrogenase; AGE: advanced glycation end-product; AR: aldose reductase; ARG: arginase; ATP: adenosine triphosphate; CPT: carnitine palmitoyltransferase; DHAP: dihydroxyacetone phosphate; eNOS: endothelial nitric oxide synthase; ETC: electron transport chain; F6P: fructose 6-phosphate; F1,6P_2_: fructose 1,6-bisphosphate; F2,6P_2_: fructose 2,6 bisphosphate; FA: fatty acid; G6P: glucose 6-phosphate; G6PD: glucose 6-phosphate dehydrogenase; GAPDH: glyceraldehyde 3-phosphate dehydrogenase; GFAT: glutamine-6-phosphate amidotransferase; GlucN6P: glucosamine-6-phosphate; GLS: glutaminase; GLUT: glucose transporter; GS: glutamine synthetase; GSH: glutathione: hCYS: homocysteine; HMG-CoA: hydroxymethylglutaryl coenzyme A; IDH; isocitrate dehydrogenase; LDH: lactate dehydrogenase; MCT: monocarboxylate transporter; ME: malic enzyme; MET: methionine; meTHF: 5.10-methylene-tetrahydrofolate; mTHF: 5-methyltetrahydrofolate; MS: methionine synthetase; NAD: nicotinamide adenine dinucleotide; NADPH: nicotinamide adenine dinucleotide phosphate; NO: nitric oxide; ODC: ornithine decarboxylase; PFK1: phosphofructokinase-1 PFKFB3: 6-phosphofructo-2-kinase/fructose-2,6-bisphosphatase-3; PGK: phosphoglycerate kinase; ROS: reactive oxygen species; RPI: ribose-5-phosphate isomerase; SAH: S-adenosylhomocysteine: SAM: S-adenosylmethionine; TCA cycle: tricarboxylic acid cycle; THF: tetrahydrofolate; TKT: transketolase; UDP-GlcNAc: uridine diphosphate N-acetylglucosamine.

Despite their close proximity to oxygenated blood, ECs rely on glycolysis instead of oxidative metabolism for adenosine triphosphate (ATP) production (Parra-Bonilla *et al*, 2010; De Bock *et al*, 2013b). In fact, under physiological conditions, over 80% of ATP is produced by converting glucose into lactate (Fig 1). Less than 1% of glucose-derived pyruvate enters the mitochondria for oxidative metabolism through the tricarboxylic acid cycle (TCA) and subsequent ATP production via the electron transport chain (ETC) (Fig 1) (Culic *et al*, 1997; De Bock *et al*, 2013b). However, ECs retain the ability to switch to oxidative metabolism of glucose, amino acids and fatty acids in case of reduced glycolytic rates (Krutzfeldt *et al*, 1990; Dranka *et al*, 2010).

**Glossary**

**1C metabolism**

A complex metabolic network characterized by the transfer of carbon from serine/glycine for folate compound chemical reactions and involved in nucleotide, lipid and protein biosynthesis, redox homeostasis and production of methylation substrates.

**Advanced glycation end products (AGEs)**

Proteins or lipids that have been non-enzymatically glycated, often as a result of hyperglycemia and/or oxidative stress, that cause damaging intracellular and extracellular dysfunction.

**Angiogenesis**

Growth of new blood vessels from existing microvasculature.

**Endothelium**

Continuous inner lining of all vasculature composed of endothelial cells (ECs), which regulates physiological vascular function and angiogenesis.

**EC dysfunction**

Inability of endothelial cells to fulfill their physiological role as mediators of the blood barrier and vasotone.

**Fatty acid oxidation**

Metabolism of fatty acids in mitochondria into acetyl-CoA to fuel the TCA cycle.

**Glycolysis**

Anaerobic metabolism of glucose producing ATP and pyruvate

**Glycosylation**

A post-translational modification that enzymatically adds glycans, or oligosaccharides, to proteins and lipids.

**Hexosamine biosynthesis pathway**

Side pathway from glycolytic intermediate fructose 6-phosphate (F6P) that produces substrates for glycosylation.

**Isoprenoid**

Mevalonate pathway intermediates used for the production of cholesterol and as substrates for prenylation.

**Metabolic flux**

Flow of metabolites through a given metabolic pathway.

**Metabolic flux analysis**

Quantification of metabolic flux by tracing the fate of Isotope-labeled substrates.

**Metabolism**

The spectrum of organic and chemical cellular reactions dedicated to the production of energy and building blocks for general maintenance and functionality.

**Methylglyoxal pathway**

Glycolytic side pathway from dihydroxyacetone phosphate (DHAP) that results in production of methylglyoxal and/or AGEs.

**Oxidative metabolism**

Aerobic metabolic pathways that break down substrates through oxidation for energy production and biosynthesis.

**Pentose phosphate pathway**

Metabolic pathway important for redox homeostasis and biosynthesis which utilizes glucose-derived glucose-6-phosphate (G6P) for production of NADPH through its oxidative branch, and fructose 6-phosphate (F6P) and 3-phosphoglycerate (3PG) for nucleotide production in its non-oxidative branch.

**Polyol pathway**

Pathway implicated in diabetic endothelial dysfunction by reduction of glucose into sorbitol and then fructose to fuel production of AGEs.

**Prenylation**

Post-translational addition of isoprenoids such as farnesyl or geranyl–geranyl to a protein.

**Quiescence**

Cell state defined by a lack of activity.

**Reactive nitrogen species**

Highly reactive nitrogen-containing molecules that often interact with ROS, promote oxidative stress and reduce bioavailability of nitric oxide.

**Reactive oxygen species (ROS)**

Highly reactive molecules that contain oxygen (produced by aerobic metabolic processes) and are involved in normal cell homeostasis and signaling, but whose accumulation, termed oxidative stress, leads to cell damage.

**Stalk cell**

Endothelial cells that trail migratory tip cells and proliferate to extend growth of a new blood vessel during sprouting angiogenesis.

**Tip cell**

Migratory endothelial cells that lead spouting microvessels up a chemokine gradient during angiogenesis.

ECs lining peripheral tissue vessels or the blood brain barrier (BBB) express multiple members of the two major families of sugar transporters, that is, glucose transporters (GLUT) and sodium/glucose co-transporters (SGLTs), but the high-affinity GLUT1 is considered to be the main route of glucose uptake in ECs (Fig 1) (Mann *et al*, 2003; Gaudreault *et al*, 2004, 2008; Sahoo *et al*, 2014). Phosphorylation of intracellular glucose by hexokinase (HK) destines it for metabolic utilization, predominately by conversion to lactate via glycolysis (Fig 1) (Paik *et al*, 2005; De Bock *et al*, 2013b). Glycolytic intermediates also serve as precursors for biosynthetic pathways including the pentose phosphate pathway (PPP), hexosamine biosynthesis and glycogenesis (Fig 1, for an extensive review see (De Bock *et al*, 2013a,b)).

The PPP consists of oxidative and non-oxidative branches, and its overall flux is determined by the rate-limiting enzyme glucose-6-phosphate dehydrogenase (G6PD) (Fig 1). Partially regulated by VEGF signaling, G6PD destines glucose-6-phosphate (G6P) for utilization in the PPP (Pan *et al*, 2009). The oxidative branch of the PPP converts G6P into ribulose-5-phosphate (Ru5P) and produces NADPH from NADP^+^, thereby generating reducing power to maintain EC redox balance and biosynthetic reactions (Dobrina & Rossi, 1983; Jongkind *et al*, 1989; Spolarics & Spitzer, 1993; Spolarics & Wu, 1997; Vizan *et al*, 2009). The non-oxidative branch converts Ru5P into xylulose-5-phosphate (Xu5P) and ribose-5-phosphate (R5P), the latter is necessary for nucleotide biosynthesis (Pandolfi *et al*, 1995). However, PPP intermediates may also be converted back into glycolytic intermediates via the action of transketolase (TKT) and transaldolase. These reactions are reversible, allowing biosynthesis of macromolecules from glycolytic metabolites via the non-oxidative arm.

The hexosamine biosynthesis pathway starts with the conversion of the glycolytic intermediate fructose-6-phosphate (F6P) into glucosamine-6-phosphate (GlucN6P) (Fig 1). GlucN6P is then metabolized to uridine diphosphate N-acetylglucosamine (UDP-GlcNAc), a key substrate for glycosylation reactions that control many aspects of EC function (Benedito *et al*, 2009; Laczy *et al*, 2009; Croci *et al*, 2014). The polyol pathway and methylglyoxal pathways are glycolysis side-pathways that are mostly known for their role in cardiovascular disease (Fig 1; see below) (Goldin *et al*, 2006).

Other metabolic pathways are less well characterized in ECs. Fatty acid (FA) oxidation (FAO) and glutamine oxidation have been implicated in replenishing the TCA cycle to produce ATP via oxidative phosphorylation (Fig 1) (Leighton *et al*, 1987; Hinshaw & Burger, 1990; Dagher *et al*, 1999, 2001; De Bock *et al*, 2013b). However, since ECs predominately rely on glucose metabolism to provide ATP, the energetic function of FAO and glutamine oxidation is not clear (De Bock *et al*, 2013b). FAs and amino acids can serve as precursors for biomass production, but such a role in ECs has not been demonstrated using isotope tracer labeling studies. FAO produces significant amounts of nicotinamide adenine dinucleotide phosphate (NADPH), which is an important co-factor in many biosynthetic reactions and essential to maintain redox balance. In addition, FAO generates acetyl-coA which is another important precursor for biomolecule production.

For example, acetyl-CoA is used, among other things, for the synthesis of cholesterol via the mevalonate pathway (Fig 1). Although endothelial cholesterol metabolism has been poorly studied, perturbations in cholesterol homeostasis are known to affect key EC functions such as intracellular signaling, inflammatory activation, nitric oxide synthesis and angiogenesis (Boger *et al*, 2000; Ivashchenko *et al*, 2010; Whetzel *et al*, 2010; Xu *et al*, 2010; Fang *et al*, 2013). ECs express all the cholesterol biosynthesis enzymes and the LDL receptor for extracellular uptake (Fig 1). These proteins are under transcriptional control of the sterol regulatory element binding protein (SREBP1 and -2) and liver X receptors (LXR) (Noghero *et al*, 2012). SREBP1 and LXRs inhibit cholesterol synthesis and absorption, whereas SREBP2 induces synthesis and inhibits cholesterol efflux via transcriptional repression of the ATP-binding cassette (ABC) transporter 1 ABCA1, which together with ABCG1 mediates cholesterol efflux from ECs (Hassan *et al*, 2006). Notably, endothelial SREBP2 also controls expression of arginine metabolism enzymes, although the physiological significance of this interaction between cholesterol and arginine metabolism remains to be determined (Zeng *et al*, 2004).

Arginine and glutamine are the best studied amino acids (AAs) in ECs. Arginine is a metabolite in the ornithine cycle and converted into citruline and nitric oxide (NO) by endothelial nitric oxide synthase (eNOS) (Fig 1) (Sessa *et al*, 1990). Alterations in arginine and eNOS metabolism are among the best-characterized causes of EC dysfunction and a prime therapeutic target (Leiper & Nandi, 2011). Glutamine is the most abundant AA in the peripheral blood and preferentially taken up by ECs via the solute carrier family 1 member 5 (SLC1A5) transporter (Fig 1) (Herskowitz *et al*, 1991; Pan *et al*, 1995). Glutamine-utilizing pathways are mainly biosynthetic and can be divided into those that utilize the γ-nitrogen (nucleotide biosynthesis, hexosamine biosynthesis, asparagine synthesis) and those that use the α-nitrogen or carbon backbone (DeBerardinis & Cheng, 2010). The latter reactions use glutamine-derived glutamate rather than glutamine itself and include glutathione (GSH) synthesis, anaplerotic refueling of the TCA cycle and biosynthesis of polyamines, proline and other non-essential AAs (NEAAs) (Fig 1) (DeBerardinis & Cheng, 2010).

Serine and glycine are especially interesting examples of glutamine / glutamate-derived NEAAs, not only because of their direct effects on ECs (Weinberg *et al*, 1992; Rose *et al*, 1999; Yamashina *et al*, 2001; Mishra *et al*, 2008; den Eynden *et al*, 2009; McCarty *et al*, 2009; Stobart *et al*, 2013), but also since their synthesis requires both the glutamate α-nitrogen and the glycolytic intermediate 3-phosphoglycerate (3PG) (Fig 1) (Locasale, 2013). Hence, serine and glycine metabolism integrates metabolic input from central carbon (glycolysis) and nitrogen (glutamine) metabolism. Moreover, the reversible interconversion of serine and glycine is directly coupled to one-carbon metabolism, intermediates of which are considered important targets to treat cardiovascular disease (Fig 1; see below) (Locasale, 2013). In fact, while EC metabolism is largely understudied, several of the above-mentioned metabolic pathways have been implicated as mediators of pathological angiogenesis or EC dysfunction.

## EC metabolism in diseases characterized by angiogenesis and EC hyperproliferation

### Cancer

**Figure 2:**
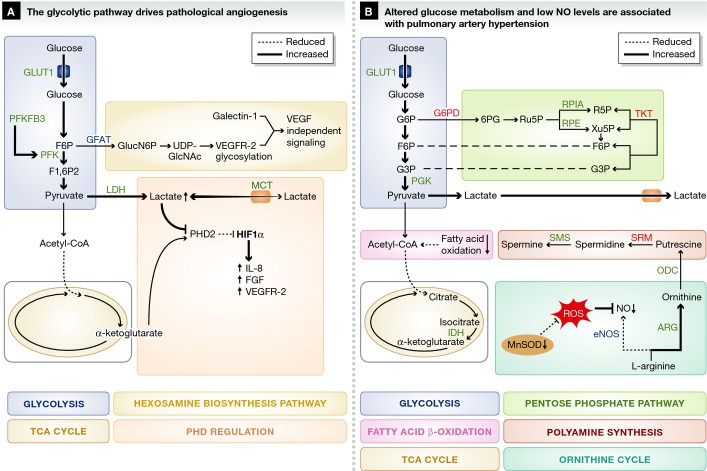
Metabolic pathways implicated in diseases characterized by pathological angiogenesis or hyperproliferative ECs (A) Angiogenic ECs rely on glycolysis, instead of oxidative metabolism, for ATP production and upregulate PFKFB3 to increase the conversion of glucose into lactate through glycolysis. Lactate is secreted and taken up through lactate transporters. High Lactate influx through MCT1 results in increased intracellular lactate levels that compete with α-ketoglutarate for PHD-2 binding, leading to HIF-1α stabilization and upregulation of pro-angiogenic genes. VEGFR-2 glycosylation is required for galectin-1-induced VEGF-independent signaling. (B) PAH ECs are metabolically characterized by high aerobic glycolysis and low oxidative metabolism. NO production through eNOS is impaired due to upregulation of arginase II and increased oxidative stress due to limited availability of MnSOD. In addition, several enzymes in the pentose phosphate pathway and polyamine biosynthesis pathway are differentially expressed in PAH ECs, but the importance of these findings remains to be determined (B). Green font / bold line: upregulated, red font / broken line: downregulated. For clarity, not all metabolites and enzymes of the depicted pathways are shown. Abbreviations: as in Fig 1. FGF: fibroblast growth factor; HIF: hypoxia-inducible factor; IL: interleukin; PHD: prolyl hydroxylase domain; R5P: ribose-5-phosphate; RPE: ribulose-5-phosphate 3-epimerase; RPIA: ribose-5-phosphate isomerase; Ru5P: ribulose-5-phosphate; SRM: spermidine synthase; VEGFR: vascular endothelial growth factor receptor; Xu5P: xylulose-5-phosphate.

Tumors need blood vessels to supply oxygen and detoxify waste products (Jain, 1987; Papetti & Herman, 2002; Welti *et al*, 2013). When tumors become too large to allow adequate diffusion of oxygen and nutrients from local vasculature they secrete pro-angiogenic growth factors to induce angiogenesis (Bergers & Benjamin, 2003). Pharmacological inhibition of growth factor signaling (primarily vascular endothelial growth factor (VEGF) signaling) is the only clinically approved anti-angiogenic strategy, but the benefits are limited as tumors acquire resistance within months after treatment initiation (Bergers & Hanahan, 2008; Carmeliet & Jain, 2011; Ebos & Kerbel, 2011; Welti *et al*, 2013). Escape from anti-angiogenic therapy is mediated by increased secretion of pro-angiogenic factors, activation of alternative angiogenic signaling pathways, recruitment of pro-angiogenic accessory cells and other mechanisms (Loges *et al*, 2010; Sennino & McDonald, 2012). A recent report indicated that glycosylation-dependent interactions of galectin-1 with VEGF receptor 2 (VEGFR2) could activate pro-angiogenic signaling even when the VEGF ligand is blocked (Fig 2A) (Croci *et al*, 2014). Hence, angiogenic signaling is robust and redundant, and inhibition of individual signaling molecules and growth factors can be overcome by escape mechanisms.

The switch from a quiescent to an angiogenic phenotype (as occurs in cancer) is metabolically demanding and mediated by adaptations in EC metabolism (Fig 2A). While the changes in metabolic fluxes of ECs, freshly isolated from tumors, have not been characterized yet, ECs in tumors and inflamed tissues likely resemble highly activated ECs. Lactate dehydrogenase B (LDH-B) is upregulated in tumor endothelium, and VEGF signaling increases glycolytic flux by inducing GLUT1 and the glycolytic enzyme 6-phosphofructo-2-kinase/fructose-2,6-bisphosphatase-3 (PFKFB3) (Fig 2A) (van Beijnum *et al*, 2006; Yeh *et al*, 2008; De Bock *et al*, 2013b). PFKFB3 catalyzes the synthesis of fructose-2,6-bisphosphate (F2,6P_2_), which is an allosteric activator of 6-phosphofructo-1-kinase (PFK-1) (Van Schaftingen *et al*, 1982). PFK-1 converts fructose-6-phosphate (F6P) to fructose-1,6-bisphosphate (F1,6P_2_) in the rate-limiting step of glycolysis. EC-specific PFKFB3 deletion diminishes retinal and hindbrain vascularization in mice, showing that increased glycolytic flux is required for growth factor-induced angiogenesis (De Bock *et al*, 2013b). Moreover, PFKFB3 overexpression in zebrafish drives EC specification into sprout forming tip cells, even in the presence of tip cell-inhibitory Notch signals that promote proliferating stalk elongating cells (De Bock *et al*, 2013b). Increased glycolysis not only provides energy for proliferation and biosynthesis, but also for locomotion. Specifically, PFKFB3 and other glycolytic enzymes co-localize with F-actin bundles in filopodia and lamellipodia to produce ATP needed for rapid actin remodeling, underlying locomotion and tip cell formation (De Bock *et al*, 2013b).

The important role of glycolysis in angiogenesis provides opportunities for therapeutic targeting. Indeed, pharmacological blockade with 3-(3-pyridinyl)-1-(4-pyridinyl)-2-propen-1-one (3PO) or EC-specific genetic silencing of PFKFB3 inhibits tumor growth *in vivo* (Xu *et al*, 2014). In addition, 3PO inhibits glycolytic flux partially and transiently and has recently shown efficacy in reducing pathological angiogenesis in a variety of disease models (Schoors *et al*, 2014b; Xu *et al*, 2014). The systemic harm caused by inhibiting glycolysis is minimal, however, showing that even moderate, short-term impairment of glycolysis renders ECs more quiescent without overt detrimental side effects (Schoors *et al*, 2014b). The finding that partial and transient reduction of glycolysis may be sufficient to inhibit pathological angiogenesis provides a paradigm shift in our thinking about anti-glycolytic therapies, away from complete and permanent blockade of glycolysis, which can induce undesired adverse systemic effects.

Aside from serving as an energy source or building blocks for biosynthesis, glycolytic metabolites can also modulate angiogenesis by acting as bona fide signaling molecules. This is evidenced by the observation that glycolytic tumor cells secrete lactate, which is taken up by ECs through the monocarboxylate transporter 1 (MCT1) (Fig 2A) (Sonveaux *et al*, 2012). Instead of being metabolized, lactate induces HIF-1α activation leading to increased expression of VEGFR2 and bFGF (Sonveaux *et al*, 2012). Moreover, lactate competes with α-ketoglutarate for binding to the oxygen sensing prolyl hydroxylase-2 (PHD-2), resulting in diminished PHD-2 activity and subsequent hypoxia-inducible factor-1α (HIF-1α) stabilization (Fig 2A). Stabilized HIF-1α induces pro-angiogenic signaling pathways such as nuclear factor kappa-light-chain-enhancer of activated B-cells (NFkB)/interleukin 8 (IL-8) leading to increased angiogenesis (Fig 2A) (Hunt *et al*, 2007; Vegran *et al*, 2011; Sonveaux *et al*, 2012). Exploratory studies found that lactate induces angiogenesis *in vivo* and that pharmacological blockade of MCT1 inhibits angiogenesis and reduces tumor growth in mice (Sonveaux *et al*, 2012). Together, these data suggest an intricate relationship between classical pro-angiogenic signals such as VEGF, HIF-1α and hypoxia, and EC glucose metabolism. Targeting EC glucose metabolism to inhibit tumor angiogenesis is in its infancy as a therapeutic strategy, but recent evidence suggests its viability.

### Pulmonary arterial hypertension

Idiopathic pulmonary arterial hypertension (PAH) is characterized by heightened pressure in pulmonary arteries caused by excessive EC proliferation and vascular dysfunction (Xu & Erzurum, 2011). Emerging evidence indicates that metabolic abnormalities underlie PAH (Fig 2B) (Sutendra & Michelakis, 2014; Zhao *et al*, 2014). In line with recent findings that glycolysis regulates angiogenesis, hyperproliferative PAH ECs rely on increased glycolytic flux and reduced oxygen consumption, which may be related to HIF-1α overexpression (Fig 2B) (Xu *et al*, 2007; Fijalkowska *et al*, 2010; Majmundar *et al*, 2010; Tuder *et al*, 2012). Human pulmonary ECs expressing mutated bone morphogenetic protein receptor 2 (BMPR2), which confers PAH, show altered expression of several glycolytic enzymes including GLUT1 and phosphoglycerate kinase 1 (PGK1). PAH ECs also show increased expression of enzymes of the PPP (R5P isomerase, Ru5P-3-epimerase) and polyamine biosynthesis pathway (ornithine decarboxylase (ODC), spermine synthase (SMS)). These metabolic changes may underlie the rapid proliferation of PAH ECs, since glycolysis, the PPP and mitogenic polyamines all promote cellular proliferation (Morrison & Seidel, 1995). However, the expression of other PPP and polyamine enzymes [G6PD, TKT, spermidine synthase (SRM)] is reduced—a finding that requires further explanation (Fig 2B) (Atkinson *et al*, 2002; Rudarakanchana *et al*, 2002; Long *et al*, 2006; Fessel *et al*, 2012). In addition, ECs isolated from EC-specific BMPR2 mutant mice show similarly increased expression of PGK1, indicating altogether that alterations in glycolysis as well as PPP likely underlie PAH (Majka *et al*, 2011).

In addition to alterations in glycolysis, idiopathic PAH ECs have fewer mitochondria and decreased mitochondrial metabolic activity (Xu *et al*, 2007). BMPR2 mutant ECs have reduced quantities of TCA cycle intermediates, reduced fatty acid oxidation and transcriptional reduction of several enzymes involved in fatty acid metabolism, including the rate-limiting enzyme of fatty acid oxidation carnitine palmitoyltransferase 1 (CPT1) (Fig 2) (Fessel *et al*, 2012). Together, these findings suggest reduced oxidative metabolism. Indeed, pharmacological inhibition of hyper-activated pyruvate dehydrogenase kinase (PDK), an enzyme that shunts glucose-derived pyruvate away from oxidative TCA metabolism, has shown therapeutic efficacy. However, whether these effects are mediated via ECs specifically remains to be determined (McMurtry *et al*, 2004). For unexplained reasons, PAH patients also show increased isocitrate dehydrogenase (IDH)-1 and IDH-2 serum activity, a finding that corroborates with the increased IDH activity observed in BPMR2 mutant ECs (Fessel *et al*, 2012). Still, the mechanisms that alter metabolic pathways in PAH ECs and the importance of some of these metabolic adaptations in the pathogenesis of PAH remain unclear.

Reduced nitric oxide (NO) levels are another hallmark of PAH ECs (Fijalkowska *et al*, 2010). Low NO levels may be related to the reduced levels of the mitochondrial antioxidant manganese superoxide dismutase (MnSOD) (Fijalkowska *et al*, 2010). Indeed, MnSOD increases NO availability by clearing superoxide anion, which inactivates NO to form peroxynitrite (Fig 2) (Masri *et al*, 2008). However, other factors likely contribute to the low NO levels in PAH ECs (Xu *et al*, 2004). Indeed, human PAH ECs express high levels of arginase II, which competes with endothelial nitric oxide synthetase (eNOS) for their common substrate L-arginine (Fig 2) (Xu *et al*, 2004). Inhibition of endothelial arginase II increases NO production *in vitro,* suggesting that arginase II can be targeted to prevent EC hyperproliferation and restore NO availability (Krotova *et al*, 2010). While the mechanisms that induce abnormal metabolic activity in PAH ECs are understudied, restoring NO may provide dual benefits in preventing excessive EC proliferation as well as restoring EC vasoactivity.

The metabolic adaptations in PAH (high glycolytic rates and reduced oxidative metabolism) are partly reminiscent of the metabolic profile of angiogenic ECs. It would be thus interesting to determine if reducing glycolysis by pharmacological blockade of PFKFB3 can reduce the hyperproliferative rate in PAH ECs. Alternatively, the beneficial effects of PDK inhibition in PAH to induce oxidative metabolism could also be beneficial to block angiogenesis by preventing the glycolytic switch in ECs. Indeed, PDK blockade with dichloroacetate inhibits angiogenesis in glioblastoma patients (Michelakis *et al*, 2010).

## EC metabolism in diseases characterized by EC dysfunction

### Diabetes

**Figure 3:**
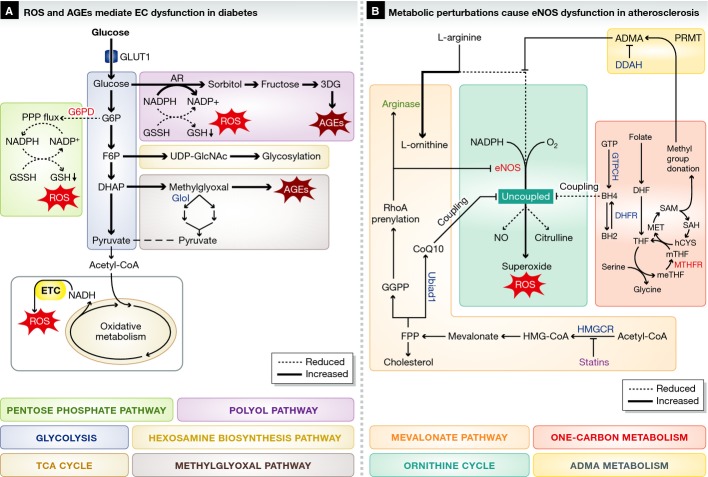
Metabolic pathways implicated in diseases characterized by EC dysfunction (A) High glucose levels in diabetes pushes glycolytic flux and cause ROS production and AGE formation. (B) Metabolic alterations that cause eNOS dysfunction mediate atherosclerosis pathogenesis. Asymmetric dimethylarginine (ADMA) competes with arginine for binding to eNOS. Arginase expression is increased and eNOS expression is decreased, leading to reduced eNOS activity. 1C metabolism and mevalonate metabolism provide eNOS coupling co-factors and inhibit ROS production. The mevalonate pathway also provides farnesyl pyrophosphate (FPP) and geranylgeranyl pyrophosphate (GGPP), required for GTPase prenylation. For clarity, not all metabolites and enzymes of the depicted pathways are shown. Green font / bold line: upregulated, red font / broken line: downregulated. Abbreviations: as in Figure 1. BH2: dihydrobiopterin; BH4: tetrahydrobiopterin; ADMA: asymmetric dimethylarginine; CoQ10: coenzyme Q10; DDAH: dimethylarginine dimethylaminohydrolase; DHF: dihydrofolate; DHFR: dihydrofolate reductase; FPP: farnesyl pyrophosphate; GGPP: geranylgeranyl pyrophosphate; GTP: Guanosine triphosphate; HMGCR: hydroxymethylglutaryl coenzyme A reductase; PRMT: protein arginine methyltransferase.

Diabetes is characterized by high blood glucose levels that affect EC metabolism and cause dysfunction (Fig 3A) (Blake & Trounce, 2013). Hyperglycemia induces peroxisome proliferator-activated receptor-gamma coactivator 1α (PGC-1α), an important regulator of metabolic gene expression and mitochondrial biogenesis (Puigserver *et al*, 1998; Herzig *et al*, 2001; Lin *et al*, 2002). PGC1α increases angiogenesis when expressed in heart and muscle cells (Arany *et al*, 2008; Patten *et al*, 2012). In contrast, diabetes-induced PGC-1α expression in ECs renders them less responsive to angiogenic factors and blunts angiogenesis (Sawada *et al*, 2014).

In addition to affecting gene expression, high glucose levels alter metabolism to induce the production of reactive oxygen species (ROS) and reactive nitrogen species (RNS), which might be mediators of EC dysfunction (Fig 3) (Blake & Trounce, 2013). High glucose levels cause ECs to produce ROS via activation of NADPH-dependent oxidases (Inoguchi *et al*, 2003). In addition, hyperglycemia inhibits PPP flux by down-regulation of G6PD, the rate-limiting enzyme of the PPP. The PPP is an important source of intracellular NADPH, which is necessary to convert oxidized glutathione (GSSH) into reduced GSH, a critical ROS scavenger (Fig 3A) (Leopold *et al*, 2003; Zhang *et al*, 2012). Therefore, by reducing PPP flux, high glucose depletes NADPH levels and contributes to ROS accumulation (Goldin *et al*, 2006). Interestingly, G6PD overexpression restores redox homeostasis in high glucose cultured ECs (Leopold *et al*, 2003; Zhang *et al*, 2012). Some studies suggest that high glucose shifts the normally glycolytic EC metabolism toward oxidative metabolism and increased mitochondrial respiration (Fig 3). However, these results appear contextual, as other studies did not report such an induction of oxidative metabolism (Nishikawa *et al*, 2000; Koziel *et al*, 2012; Pangare & Makino, 2012; Dymkowska *et al*, 2014). While the precise effects on mitochondrial respiration require further study, hyperglycemia-induced mitochondrial ROS induces DNA breaks and thereby activates polyAPD-ribose polymerase (PARP-1) (Du *et al*, 2000, 2003; Nishikawa *et al*, 2000; Giacco & Brownlee, 2010; Blake & Trounce, 2013). PolyADP-ribosylation by PARP-1 inactivates GAPDH and stalls glycolysis, allowing accumulation of glycolytic metabolites (Du *et al*, 2003).

Accumulation of F6P increases the flux through the hexosamine biosynthesis pathway (HBP), which produces UDP-GlcNac, an important precursor of glycosylation reactions (Fig 3A) (Brownlee, 2001). While glycosylation is important for physiological EC function, hyperglycemia-induced protein glycosylation inhibits angiogenic functions (Du *et al*, 2001; Federici *et al*, 2002; Luo *et al*, 2008). Other glycolytic intermediates are diverted into the polyol and methylglyoxal pathways that produce damaging agents such as ROS and advanced glycation end products (AGEs) (Fig 3A) (Goldin *et al*, 2006). AGEs induce vascular dysfunction by altering extracellular matrix protein function and dysregulating cytokine expression (Yan *et al*, 2008). In addition, receptor of AGE (RAGE) binding by AGEs in vascular cells causes inflammation and reduced NO availability associated with vascular complications in diabetic patients (Bucala *et al*, 1991; Vlassara *et al*, 1995; Min *et al*, 1999; Wautier & Schmidt, 2004; Goldin *et al*, 2006; Manigrasso *et al*, 2014).

Excess glucose that cannot be metabolized by glycolysis enters the polyol pathway when converted into sorbitol by aldose reductase (AR) at the expense of NADPH, increasing ROS. Sorbitol is subsequently converted into fructose and the highly reactive 3-deoxyglucosone (3DG), which promotes the formation of AGEs (Fig 3A) (Kashiwagi *et al*, 1994; Oyama *et al*, 2006; Giacco & Brownlee, 2010; Sena *et al*, 2012; Yoshida *et al*, 2012). Transgenic overexpression of human AR in the Gendothelium of diabetic mice accelerates atherosclerosis formation and inhibition of endothelial AR reduces intracellular ROS, EC migration and proliferation (Obrosova *et al*, 2003; Tammali *et al*, 2011; Vedantham *et al*, 2011; Yadav *et al*, 2012). Methylglyoxal is another AGE precursor and produced from the glycolytic intermediates glyceraldehyde-3-phosphate (G3P) and dihydroxyacetone phosphate (DHAP). Methylglyoxal is detoxified by conversion into pyruvate via the multienzyme glyoxalase system, of which glyoxalase-I (GloI) is rate-limiting (Fig 3A) (Thornalley, 1993). Glyoxalase-I overexpression reverses hyperglycemia-induced angiogenesis defects *in vitro* and transgenic overexpression of glyoxalase-I in rats reduces vascular AGE formation and improves vasoreactivity (Brouwers *et al*, 2010, 2014) (Ahmed *et al*, 2008). Together, these observations indicate that targeting AR and glyoxalase might confer a therapeutic benefit in diabetic patients.

### Atherosclerosis

Atherosclerosis is a chronic inflammatory process in the blood vessel wall leading to luminal narrowing and subsequent cardiovascular events (Hopkins, 2013). Systemic metabolic perturbations are among the most important risk factors of atherosclerosis. However, metabolic flux changes have not been studied in ECs isolated from atherosclerotic lesions, and the effects of atherosclerosis on central metabolism of ECs thus remains to be characterized. Nonetheless, EC metabolism is strongly associated with a key pathophysiological feature of atherosclerosis: reduced and uncoupled eNOS activity resulting in low NO bioavailability and high ROS production (Fig 3B) (Kawashima & Yokoyama, 2004). eNOS activity critically depends on the availability of L-arginine, co-factor tetrahydrobiopterin (BH4) (Fig 3B) and possibly co-enzyme Q10 (CoQ10) (Gorren *et al*, 2000; Crabtree *et al*, 2009a; Mugoni *et al*, 2013). If L-arginine, BH4 or CoQ10 become limited, eNOS no longer oxidizes L-arginine to form citrulline and NO, but instead produces ROS (a condition termed eNOS uncoupling) (Fig 3B) (Stroes *et al*, 1998; Mugoni *et al*, 2013). Targeting L-arginine and BH4 metabolism to increase eNOS activity in patients with cardiovascular disease is potentially beneficial, but available evidence indicates that the picture is more complex than initially anticipated.

Small-scale clinical trials indicate that administration of L-arginine to patients with coronary heart disease improves vasoresponsiveness, possibly by increasing NO production by eNOS (Lerman *et al*, 1998). Interestingly, however, intracellular and plasma arginine levels are sufficiently high to support NO biosynthesis via eNOS. Therefore, the benefits of L-arginine supplementation on elevating NO levels are not readily explained by increasing the supply of L-arginine; however, it is possible that L-arginine is compartmentalized in poorly interchangeable pools. Another possible explanation of the beneficial effects of L-arginine is competition with asymmetric methylated arginines, which bind and inhibit eNOS (Fig 3B) (Boger, 2004; Chen *et al*, 2013). More in detail, post-translational methylation of arginine residues in proteins by protein arginine methyltransferase (PRMT) results in the addition of up to two methyl groups to arginine. Protein turnover releases these post-translationally modified amino acids as asymmetric dimethylarginine (ADMA) and symmetric dimethylarginine (SDMA). The asymmetric dimethylarginines bind and uncouple eNOS resulting in increased ROS production and reduced NO availability (Fig 3B) (Dhillon *et al*, 2003; Leiper & Nandi, 2011). Hence by competing with ADMAs, supplemented L-arginine could maintain eNOS activity to produce NO (Bode-Boger *et al*, 2003). Additional potential interventions to reduce eNOS inhibition by ADMA include PRMT inhibition (to reduce arginine methylation) and activation of methylarginine catabolism by dimethylarginine dimethylaminohydrolase (DDAH) (Fig 3B) (Leiper & Nandi, 2011). Interestingly, DDAH1 is predominantly expressed in ECs and EC-specific deletion attenuates NO production and induces hypertension, indicating that ADMA scavenging by ECs is important to maintain homeostasis (Hu *et al*, 2009).

Because L-arginine is a substrate for both eNOS and arginase (Wu & Meininger, 1995), NO production depends on the relative expression levels of each enzyme (Fig 3) (Chang *et al*, 1998; Ming *et al*, 2004; Ryoo *et al*, 2008). Endothelial arginase expression is induced by many risk factors for cardiovascular disease, while reducing arginase expression restores NO production *in vitro* and improves vasodilatation *in vivo* (Ryoo *et al*, 2006, 2008; Thengchaisri *et al*, 2006; Romero *et al*, 2008). The activity of eNOS and arginase is regulated by the RhoA/ROCK signaling cascade. RhoA and Rock decrease eNOS expression, while RhoA also increases arginase activity (Fig 3B) (Laufs *et al*, 1998; Takemoto *et al*, 2002). For proper activation and localization to the cell membrane, RhoA must be prenylated (more specifically, geranylgeranylated) by geranylgeranyltransferase (GGT) using geranylgeranyl pyrophosphate (GGPP) as a substrate (Laufs & Liao, 1998). This isoprenoid is an intermediate of the mevalonate pathway, which produces cholesterol from acetyl-coA (Fig 3B). Blocking the mevalonate pathway by inhibiting HMG-coA reductase using statins lowers cholesterol synthesis and is clinically approved to prevent cardiovascular events in dyslipidemia patients. In addition, HMG-coA blockade also decreases geranylgeranyl production, which reduces RhoA activity and restores a more beneficial eNOS/arginase balance (Goldstein & Brown, 1990; Liao & Laufs, 2005). Interestingly, UBIAD1 was recently identified as a novel prenyltransferase that produces non-mitochondrial CoQ10 from farnesyl pyrophosphate (FPP), another isoprenoid produced in the mevalonate pathway (Fig 3) (Mugoni *et al*, 2013). CoQ10 is an important anti-oxidant with beneficial effects on EC function and hypothesized to be a novel co-factor required for eNOS coupling (Gao *et al*, 2012; Mugoni *et al*, 2013). Hence, in contrast to the above-mentioned beneficial effects, HMG-coA reductase inhibition might thus also have a less favorable effect by increasing ROS levels through reducing CoQ10 synthesis (Fig 3) (Mugoni *et al*, 2013).

In addition to CoQ10, eNOS requires BH4 as a co-factor. Reduced BH4 availability is found in patients at risk of atherosclerosis and promotes ROS production through eNOS uncoupling (Fig 3B) (Pieper, 1997; Stroes *et al*, 1997; Heitzer *et al*, 2000). Endothelial BH4 levels are maintained by *de novo* biosynthesis via the rate-limiting enzyme guanosine triphosphate cyclohydrolase I (GTPCH) and by a salvage pathway from dihydrobiopterin (BH2) via dihydrofolate reductase (DHFR) (Fig 3B) (Bendall *et al*, 2014). Insufficient levels of GTPCH and DHFR, important enzymes in GTP and folate metabolism, respectively, have been associated with reduced BH4 availability, endothelial dysfunction and cardiovascular disease in several preclinical models (Chalupsky & Cai, 2005; Crabtree *et al*, 2009b, 2011; Sugiyama *et al*, 2009; Kidokoro *et al*, 2013). Interestingly, DHFR not only regenerates active BH4 from oxidized inactive BH2 but is also a key enzyme in folate and one-carbon metabolism, intermediates of which in turn regulate BH4 biosynthesis and are associated with cardiovascular disease (Humphrey *et al*, 2008).

One-carbon (1C) metabolism centers around the ability of folate-derived co-enzymes to carry activated 1C units (Fig 3) (Tibbetts & Appling, 2010). DHFR catalyzes the formation of tetrahydrofolate (THF) from folate fueling 1C metabolism. THF accepts 1C units from serine to produce 5,10-methylene-THF (meTHF) and glycine. MeTHF is reduced to 5-methyl-THF (mTHF) by methylenetetrahydrofolate reductase (MTHFR) (Fig 3). Importantly, inactivating mutations in the MTHFR gene result in hyperhomocysteinemia, which decreases GTPCH and DHFR levels and may subsequently reduce BH4 levels (Bendall *et al*, 2014). Indeed, MTHFR mutations have been associated with cardiovascular disease, but the exact association is still controversial (Kelly *et al*, 2002; Klerk *et al*, 2002; Frederiksen *et al*, 2004; Yang *et al*, 2012). mTHF produced by MTHFR activity is required as a methyl donor in the methionine synthase (MS) catalyzed reaction that converts mTHF into THF (completing the folate cycle) and forms methionine (MET) from homocysteine (hCYS) (Fig 3B) (Locasale, 2013). Methionine is used to generate S-adenosylmethionine (SAM), which is an important methyl donor and plays a pivotal role in methylation of lysine and arginine residues in proteins (Fig 3B) (Leiper & Nandi, 2011). As discussed above, methylated arginine residues are emerging as important mediators of EC dysfunction. Moreover, SAM-mediated protein methylation produces S-adenosylhomocysteine, which is converted back into homocysteine. Homocysteine decreases the bioavailability of BH4 possibly through downregulation of GTPCH and DHFR, while BH4 supplementation alleviates homocysteine-induced EC dysfunction (Dhillon *et al*, 2003; Topal *et al*, 2004). Together, these findings suggest that dysregulation of endothelial 1C metabolism is involved in the pathogenesis of cardiovascular disease, but the exact mechanisms remain to be elucidated. Nonetheless, early clinical and preclinical studies have found that therapeutic targeting of 1C metabolism, for example, via folate supplementation lowers levels of homocysteinemia and increases BH4 regeneration from BH2 (Verhaar *et al*, 2002). However, large-scale clinical trials failed to show benefits of folate or BH4 supplementation to prevent cardiovascular disease (Clarke *et al*, 2010; Cunnington *et al*, 2012; Marti-Carvajal *et al*, 2013). These clinical and preclinical findings suggest that while L-arginine, folate, methionine, COQ10 and homocysteine metabolism are potential therapeutic targets, a more detailed understanding of how these pathways cause dysfunction is required to design more rational therapeutic agents.

## EC metabolism in the pathogenesis of other diseases

EC metabolism is best characterized in the diseases discussed above. However, these represent only a minor fraction of the disorders in which pathological EC responses are presumably involved. Indeed, it is highly likely that EC metabolic alterations are also involved in the pathogenesis of other diseases such as ischemia, pre-eclampsia, vasculitis, vascular neoplasms and others although this has hardly been studied.

On the other hand, many of the EC metabolic alterations that lead to EC dysfunction are likely induced by cardiovascular risk factors such as those that characterize metabolic syndrome, hyperhomocysteinemia and hyperuricemia. For example, elevated serum uric acid (a breakdown product of purine nucleotides generated by xanthine oxidase with potent anti-oxidant activity) is common in patients with hypertension and may even be a root cause of EC dysfunction leading to cardiovascular disease (Feig *et al*, 2008). Interestingly, while uric acid has been described as major anti-oxidant in human plasma, ECs exposed to uric acid display increased ROS production creating a paradox that has not been resolved (Lippi *et al*, 2008; Sautin & Johnson, 2008). Regardless, in cardiovascular disease models uric acid reduces mitochondrial content, intracellular ATP and arginase activity (Zharikov *et al*, 2008; Sanchez-Lozada *et al*, 2012). In addition, uric acid inhibits NO production in ECs *in vitro*, and *in vivo* levels of serum nitrites (an indicator of NO production) are inversely proportional to serum uric acid concentrations (Khosla *et al*, 2005). Interestingly, ECs exposed to uric acid increase expression of AR and alter expression of several other proteins linked to metabolism (Zhang *et al*, 2014). These studies suggest that hyperuricemia induces EC dysfunction through metabolic alterations. Whether the same is true for other cardiovascular risk factors remains in question.

A broader characterization of EC metabolism in the future might reveal novel therapeutic targets in metabolic pathways that are generally not considered to be important in pathological EC function. Recent findings that endothelial cholesterol efflux to high-density lipoprotein regulates angiogenesis (Fang *et al*, 2013), and that EC-specific insulin receptor knock-out accelerates atherosclerotic plaque formation (Gage *et al*, 2013) point to a key role for EC metabolism in the pathogenesis of disease and indicate that many more yet to be identified non-traditional but potentially druggable metabolic enzymes, transporters and pathways may play a role in vascular disease.

**Pending issues**

The findings in this review suggest that blood vessel pathology is mediated, or at least characterized, by disease-specific alterations. However, at present, there are no studies that incorporate state-of-the-art metabolomics tools to characterize EC metabolism in disease. Metabolic profiling using isotope incorporation studies and metabolic flux analysis could greatly increase our understanding of the metabolic alterations that underlie EC pathology.

*In vivo* studies to characterize EC metabolism in animal models of human disease could provide highly relevant insight in disease-specific metabolic alterations. However, this requires isolation of ECs from diseased tissue, which at present poses technical and interpretational challenges for proper analysis of metabolism using advanced metabolomics methods.

Another pressing issue is the lack of studies characterizing metabolism in patient-derived tissue using either *in* or *ex* vivo models. The recent development of new protocols to isolate ECs from patient tissue offers the possibility to study metabolism in clinically relevant systems. Accordingly, such studies could greatly advance the identification of novel biomarkers and therapeutic targets in EC metabolism.

## Therapeutic targeting of EC metabolism

**Figure 4:**
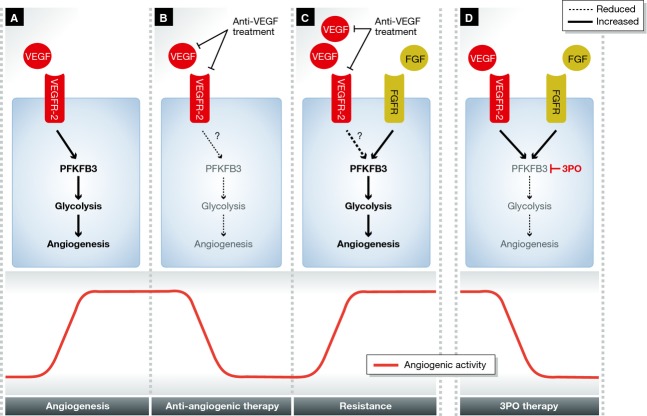
Targeting EC metabolism as an alternative to targeting growth factors in angiogenesis (A) Vascular endothelial growth factor (VEGF) induces 6-phosphofructo-2-kinase/fructose-2,6-bisphosphatase-3 (PFKFB3) and increases glycolytic flux, required for angiogenesis. (B) Anti-VEGF treatment reduces glycolytic flux and angiogenesis. (C) Increased growth factor signaling through alternative pathways, in this case fibroblast growth factor (FGF), mediates resistance to anti-angiogenic therapy. (D) Pharmacological targeting of PFKFB3 with (3PO) reduces angiogenesis irrespective of growth factor signaling and is therefore possibly less prone to resistance. Abbreviations: as in Figure 1. 3PO: 3-(3-pyridinyl)-1-(4-pyridinyl)-2-propen-1-one; FGF: fibroblast growth factor.

Overall, it is clear that pathological blood vessel responses are associated with metabolic alterations in ECs. These metabolic adaptations are not just innocent bystanders, but in many cases mediate important aspects of disease. Increased EC glucose metabolism is emerging as a key feature of angiogenic and hyper-proliferative ECs. Targeting EC glucose metabolism has recently been shown as a viable strategy to curb pathological angiogenesis, but is still in its infancy (Schoors *et al*, 2014b). Recent technical and conceptual advances, however, now make it possible to perform comprehensive metabolic studies. These technical breakthroughs have led to a resurgent interest in targeting cell metabolism for therapeutic gains. As a proof of concept, targeting EC metabolism by pharmacological inhibition of the glycolytic enzyme PFKFB3 has shown recent success in inhibiting pathological angiogenesis (Fig 4) (De Bock *et al*, 2013b; Schoors *et al*, 2014b; Xu *et al*, 2014). These results, together with the observation that EC metabolism is altered in many diseases, suggest that EC metabolism is an attractive and viable but understudied therapeutic target.

## For more information

Author website: http://www.vrc-lab.be/peter-carmeliet
